# Glycan Mimetics from Natural Products: New Therapeutic Opportunities for Neurodegenerative Disease

**DOI:** 10.3390/molecules24244604

**Published:** 2019-12-16

**Authors:** Wenyue Wang, Sandeep Gopal, Roger Pocock, Zhicheng Xiao

**Affiliations:** Development and Stem Cells Program, Monash Biomedicine Discovery Institute and Department of Anatomy and Developmental Biology, Monash University, Melbourne, Victoria 3800, Australia; wenyue.wang@monash.edu (W.W.); sandeep.gopal@monash.edu (S.G.)

**Keywords:** glycans, neurodegenerative disease, glycomimetics, natural products, therapeutic

## Abstract

Neurodegenerative diseases (NDs) affect millions of people worldwide. Characterized by the functional loss and death of neurons, NDs lead to symptoms (dementia and seizures) that affect the daily lives of patients. In spite of extensive research into NDs, the number of approved drugs for their treatment remains limited. There is therefore an urgent need to develop new approaches for the prevention and treatment of NDs. Glycans (carbohydrate chains) are ubiquitous, abundant, and structural complex natural biopolymers. Glycans often covalently attach to proteins and lipids to regulate cellular recognition, adhesion, and signaling. The importance of glycans in both the developing and mature nervous system is well characterized. Moreover, glycan dysregulation has been observed in NDs such as Alzheimer’s disease (AD), Huntington’s disease (HD), Parkinson’s disease (PD), multiple sclerosis (MS), and amyotrophic lateral sclerosis (ALS). Therefore, glycans are promising but underexploited therapeutic targets. In this review, we summarize the current understanding of glycans in NDs. We also discuss a number of natural products that functionally mimic glycans to protect neurons, which therefore represent promising new therapeutic approaches for patients with NDs.

## 1. Introduction

Glycans are ubiquitous across the natural world and can be found in both prokaryotes and eukaryotes. Glycans are carbohydrate chains (monosaccharides, oligosaccharides, or polysaccharides) that either exist in a free state or attached to proteins and lipids [1]. Most glycans are located on the cell surface and participate in cell adhesion, signal transduction, and structural maintenance of cells and tissues [2]. Some heavily glycosylated proteins, known as proteoglycans, can be found in the nucleus and cytoplasm, where they function as regulatory switches [3].

The structures of glycans are considerably diverse and complex. The base glycan structure comprises monomeric residues (monosaccharides) containing five or six carbon rings, although other more complicated monosaccharides also exist [4]. The monosaccharide residues link to each other through covalent glycosidic bonds in multiple configurations to form oligosaccharides and polysaccharides. For example, the hydroxyl group of one monosaccharide residue can potentially bind to any anomeric carbon of another monosaccharide residue to form glycosidic bonds [4]. Further, various glycosidic bond configurations based on the stereochemistry of the anomeric carbon result in diverse biological functions [5]. In addition, certain complex monosaccharides are themselves essential for specific biological functions. For instance, sialic acid, a nine-carbon sugar neuraminic acid, shows the highest concentration in the brain of humans and plays an essential role in neurotransmission [6].

## 2. Glycan Biosynthesis and Function

Glycosyltransferases (GTs) and glycosidases regulate the assembly and processing of glycans [7]. GTs are a large family of enzymes that are responsible for transferring sugar residues from glycosyl donor substrates to acceptor substrates with a high degree of substrate specificity. However, in some cases, a single GT can catalyze several reactions, or several GTs can use the same acceptor substrate [7]. In contrast, glycosidases specifically remove sugar residues from the nascent glycan or its mature form. Glycosidases are also involved in the degradation of glycans, therefore playing important roles in the metabolism of sugars [8]. Although the sequence of sugar addition and deletion is an ordered process, the biosynthesis of glycan is not a template-driven process [9], which contributes to the ability to form complex glycan structures.

Proteins or lipids attached to glycans via glycosidic bonds are known as glycoproteins and glycolipids, which represent the majority of membrane molecules. Dynamic changes in glycoproteins and glycolipids regulate cell membrane stability and cellular recognition, which is crucial for cell–cell interactions and immune responses in both physiological and pathological conditions [10,11].

The term “glycosylation” generally refers to the process of attaching glycans to lipids and proteins through multiple enzymatic processes. Glycosylation of protein is one of the most essential post-translational modifications (PTMs) [12]. There are three types of protein glycosylation: *N*-linked glycosylation (asparagine (Asn)-linked), *O*-linked glycosylation (serine (Ser)/threonine (Thr)-linked), and glycosaminoglycan attachment to proteins, forming proteoglycans [13,14,15]. Other types of protein glycosylation events are observed on lysine, tryptophan, and tyrosine residues of specific proteins, such as glycogen [16]. Correct folding and assembly of glycoproteins are essential for their proper functions, and defects in protein glycosylation pathways cause a wild range of diseases [13] including cancer [17], autoimmune disease [18], and neurodegenerative diseases (NDs) [19,20]. Glycosylation reactions occur in multiple subcellular locations, including the endoplasmic reticulum (ER), the Golgi apparatus, cytosol, and the sarcolemmal membrane [21]. In addition, the degradation of immature or misfolded glycoproteins can also occur in the ER by the ER-associated degradation (ERAD) pathway [22]. During ERAD, misfolded proteins are recognized and retrotranslocated to the cytoplasm, where they are degraded by the ubiquitin–proteasome pathway [23]. 

Non-enzymatic reactions between sugars, such as glucose and fructose, and proteins, lipids or nucleic acids, are known as glycations. Glycation is a spontaneous age-dependent posttranslational modification which leads to advanced glycation end-products (AGEs) [24]. AGEs are involved in several pathologic conditions. For example, accumulation of AGEs was observed in diabetes due to the oxidative stress and hyperglycemia [25]. Prominent AGEs include *N*-carboxymethyl-lysine (CML), pentosidine, and glucosepane [26]. Moreover, receptor for advanced glycation end-products (RAGE), the best-characterized AGE receptor, induces the generation of free radicals and the expression of inflammatory mediators [27]. Furthermore, several hallmark proteins of NDs are glycated, such as amyloid β [28], tau [29], and α-synuclein [30]. Besides, the level of glycation is positively correlated with the development of pathologies of the diseases [31]. 

### 2.1. N-linked Glycosylation

*N*-linked glycosylation of proteins regulates multiple protein functions, including protein folding and oligomerization, protein stability, and dynamic localization [32]. Numerous studies in different organisms, from yeast to mammals, have shown that *N*-linked glycosylation is essential for life [14,33,34]. *N*-glycosylation is highly organized: a typical *N*-glycosylation reaction begins with transferring the core structure of *N*-glycan in the ER, which is a precursor oligosaccharide containing 14 residue units (2 *N*-acetylglucosamine (GlcNAc), three glucose and nine mannose units). Then, the attachment of oligosaccharide to the side chains of asparagine occurs within the Asn-X-Ser/Thr sequence, followed by multiple different and sequential GT and glycosidase reactions in the ER lumen and the Golgi apparatus [35,36]. Other aspects of *N*-glycosylation reactions including removal of glucose or mannose residues or the addition of phosphate or acetyl groups onto glycans [37]. These diverse substrates and modifications contribute to the structural diversity of *N*-glycans. 

### 2.2. O-linked Glycosylation 

*O*-linked glycosylation of proteins is important in a number of bioprocesses including cellular metabolism. Dysregulated *O*-glycosylation has been observed in many disorders, including cancer [38], diabetes [39], and especially NDs [40]. Unlike *N*-glycosylation, which is mostly based on the typical core structure, *O*-glycosylation reactions are more complicated due to the multiple alternative *O*-glycan core structures that exist. In general, *O*-glycosylation involves the addition of carbohydrate chains to the oxygen atom of Thr or Ser residues by covalent linkage [41]. Among the different types of *O*-glycosylation, *O*-mannosylation and *O*-GlcNAcylation play essential roles in the nervous system [42]. 

*O*-mannose glycans are a family of highly heterogeneous, complex glycans, accounting for up to 30% of total *O*-glycan in the brain [43]. To form an *O*-mannose glycan protein, the most critical step is the formation of disaccharide, where *O*-mannose is added to Ser or Thr and is followed by the addition of any four sugars, including *N*-acetylgalactosamine (GalNAc), galactose, fucose, and sialic acid. Further modifications occur in the Golgi. For instance, GlcNAc, galactose, and sialic acid can be sequentially added to disaccharides to generate linear or multi-branched chains [44]. Some *O*-mannose-based structures can be quite complex; for example, some contain two GalNAc residues as a branch on the linkage mannose residue, while some carry an unusual 3-O-sulfated glucuronic acid called human natural killer-1 glycan antigen (HNK-1), which is implicated in neuronal cell adhesion [45]. In addition, the absence of *O*-mannose glycan leads to various muscle–eye–brain diseases (MEBs) such as congenital muscular dystrophy (CMD) [46,47].

*O*-GlcNAcylation is another major *O*-glycosylation presents in the brain [48]. In general, *O*-linked β-*N*-acetylglucosamine (*O*-GlcNAc) is attached to threonine and serine residues to form the core structure of *O*-GlcNAcylation and no other sugars are further added. In contrast to other *O*-glycosylations, *O*-GlcNAcylation biosynthesis usually occurs in the cytoplasm but is a dynamic process. For example, *O*-GlcNAc can be dynamically attached or removed from a protein by *O*-GlcNAcase (OGA) and *O*-GlcNAc transferase (OGT), suggesting a complicated relationship between these modifications that may affect various cellular functions [49]. There is an extensive body of evidence demonstrating a critical role for *O*-GlcNAcylation in multiple NDs, including Alzheimer’s disease (AD) [50], Parkinson’s disease (PD) [51], Huntington’s disease (HD), multiple sclerosis (MS), and amyotrophic lateral sclerosis (ALS) [52]. Indeed, many of the molecules playing critical roles in these NDs are *O*-GlcNAcylased. For instance, enhanced *O*-GlcNAcylation can decrease the secretion of amyloid-β (Aβ), a hallmark of AD and considered to be the initial event in disease development [50]. Further, *O*-GlcNAcylation of Tau, another hallmark protein in AD, attenuates the hyperphosphorylation of Tau, suggesting a neuroprotective role for brain *O*-GlcNAc and a potential avenue for AD treatment [50].

### 2.3. Attachment of Glycans to Lipids

Glycans can also bind to lipids. Gangliosides are one of the most abundant glycolipids in the nervous system [53], belonging to a class of sialic acid-containing glycosphingolipids (GSLs). Most gangliosides are synthesized from lactosylceramide (LacCer). For example, monosialodihexosylganglioside (GM3), a simple ganglioside, is synthesized by adding sialic acid to LacCer, which further serves as a precursor of more complex gangliosides [54]. Gangliosides play an essential role in cell recognition, cell adhesion, and signal transduction. Dynamic ganglioside expression changes have been observed during neuronal development, suggesting that specific gangliosides may need to be expressed at particular neurodevelopmental stages [53]. This hypothesis is further supported by recent studies showing that ganglioside loss leads to defective neuronal development [55]. In transgenic mice lacking all major gangliosides (sialyltransferase(ST)-I knockout mice), developmental deficits were observed in the peripheral nervous system (PNS) [54]. Furthermore, administration of monosialotetrahexosylganglioside (GM1) ganglioside can ameliorate nervous system damage and mitigate the effects of a variety of neurodegenerative processes [56].

## 3. Glycans in Neurodegenerative Diseases

Glycosylation within the central nervous system (CNS) is vital for maintaining normal brain functions. Many glycan-rich molecules in the brain are involved in neural functions, such as neuronal development, migration, and regeneration [2,6,57,58]. Dysregulated glycans have been observed in several CNS diseases, especially neurodegenerative disorders (Table 1).

Some neuron-associated glycans have been identified as biomarkers and serve as potential targets for therapy in NDs. HNK-1 antigen is abundantly expressed in the hippocampal region and regulates synaptic plasticity, neurogenesis, spatial learning and memory [59]. HNK-1 glycan also regulates immature oligodendrocyte differentiation and re-myelination in MS [60]. Polysialic acid (PSA), another important CNS glycan, is widely expressed during embryonic and postnatal brain development [6]. PSA is expressed in hippocampal neurons during synapse formation to enhance cell migration and axon pathfinding and promotes nervous system repair or regeneration. Deletion of polysialic acid imbalances excitatory and inhibitory synaptic inputs and affects the structural plasticity of interneurons [61]. These results suggest that polysialic acid might be a useful biomarker for targeting CNS diseases. Glycan expression is also associated with the innate immune response and neuroinflammation driven by microglia. Variation in glycan expression has also been detected in multiple CNS diseases, including AD, PD, HD, MS, ALS, and brain cancers [57].

### 3.1. Glycans and Alzheimer’s Disease 

As one of the most common NDs, AD affects at least 30 million people worldwide [62]. The two major pathological hallmarks of AD are deposition of Aβ peptide in the brain and intracellular aggregates of the hyperphosphorylated microtubule-associated protein tau [63].

Aβ deposition has been considered as the initial event in AD development since its discovery [64,65,66]. Although the results of clinical trials evaluating the efficacy of targeting Aβ have so far proven disappointing, the formation of neurotoxic aggregates by Aβ polymerization is still believed to be a crucial event to cause AD [67]. Aβ is generated by secretase-mediated two-step cleavage of amyloid precursor protein (APP) [68]. APP can be cleaved via several pathways, some of which lead to the formation of the Aβ peptide. In the non-amyloidogenic pathway, APP is proteolyzed by α-secretase and then γ-secretase to produce sAPPα, and three C-terminal fragments (CTF, p3 peptide, and AICD) [69]. In the amyloidogenic processing pathway, β-secretases and γ-secretases cleave APP to produce sAPPβ, C-terminal fragments including CTF 89 and CTF 99, and Aβs [69]. Among these, Aβ aggregates oligomerize, fibrillate, and finally cause AD pathology. β-site APP-cleaving enzyme 1 (BACE1) is the major β-secretase, while γ-secretase forms the assembly of four different proteins: presenilin (PS), PS enhancer 2, nicastrin, and anterior pharynx-defective 1. APP cleaved by γ-secretase occurs within the transmembrane region, producing different Aβ variants. Among these fragments, Aβ40 is the main product and Aβ42 is the most toxic product [70]. 

There is now evidence supporting a possible link between glycans and AD. AD patients have abnormal glycan profiles, with a ~40% increase in bisecting GlcNAc (a unique structural feature of *N*-glycan) observed in AD patients [71]. Similarly, soluble sialyltransferase activity in serum was significantly decreased in 12 AD patients compared to 12 age-matched controls [72]. Moreover, lectin blotting analyses of cerebrospinal fluid (CSF) proteins from non-AD, probable AD, and AD patients also showed differential sialylation in AD patients [73]. In addition, increased mRNA expression of GlcNAc transferase III, one of the responsible enzymes, was observed in AD brains [74]. Various key players in regulating AD, including APP, tau protein, BACE1, and the γ-secretase subunit nicastrin are modulated by glycosylation, and the glycosylation pattern of these proteins is also altered in AD. For example, nicastrin can be *O*-GlcNAcylated and defects of this glycosylation decreased the amount of Aβ plaques [50].

Glycans affect Aβ production: APP can be either *N*-glycosylated or *O*-glycosylated. There are two potential *N*-glycosylation sites in APP: Asn496 and Asn467 [75]. In vivo studies showed attenuating the formation of *N*-glycan reduced glycoprotein translocation to the synaptic membranes, including APP [74]. Treatment with mannosidase inhibitors blocked the formation of complex glycans, which in turn decreased APP secretion [74]. Mutations in the *N*-glycan-binding domain of APP prevented the proper secretion of APP and axonal sorting [76]. Consistent with these data, an increase in sialylation of the *N*-linked glycans of APP enhanced the secretion and metabolites of APP, with an increase in bisecting GlcNAc of APP [77]. 

Several types of *O*-glycosylation on APP have shown to affect its function. *O*-glycosylation has been detected at Thr291, Thr292, Thr576 sites in APP695, while other *O*-glycosylation sites in APP770 have also been identified in human CSF: Ser597, Ser606, Ser662, Ser611, Ser680, Thr616, Thr634, and Thr635 [78]. *O*-glycans also regulate APP processing: *O*-glycosylated APP is preferentially secreted by α-secretase, increasing levels of sAPPα and decreasing Aβ secretion [77]. Furthermore, *O*-glycosylated Aβ1-19 peptide was observed in human CSF and is increased in AD patients [79]. Insulin-degrading enzyme and neprilysin are two enzymes that critical for the clearance of Aβ [80]. However, the degradation function of these enzymes was determined by *O*-glycosylation status of their substrates. Studies showed that site-specific *O*-glycosylation shields bioactive atrial natriuretic peptide (ANP) from proteolytic degradation by insulin-degrading enzyme and neprilysin [81]. In addition, an increase in tyrosine-linked glycan on Aβ fragments has been identified in the CSF samples of AD patients and was specifically found on short Aβ 1–15 and Aβ 1–20 [82].

APP cleaved by BACE1 is the rate-limiting step in Aβ production. During maturation, BACE1 undergoes several post/co-translational modifications including *N*-glycosylation. Clinical studies have shown that increased GlcNAc on BACE1 was observed in AD patient brains compared to controls [83]. In addition, the modification of BACE-1 with bisecting GlcNAc reduces the degradation of BACE-1 in the lysosome, which leads to increased Aβ formation [83]. Interestingly, in AD pathogenesis, Aβ deposition increased oxidative stress which, in turn, upregulated the production of bisecting GlcNAc on BACE1 and prevented BACE1 degradation [84]. It therefore appears that a cycle exists between BACE1 induction and Aβ generation in AD pathology.

Glycan effects tau pathology: *N*-glycosylated tau has been detected at high levels in AD patients compared to healthy controls. Human tau contains three potential *N*-glycosylation sites: Asn359-Ile-Thr, Asn167-Ala-Thr, and Asn410-Val-Ser [85]. The glycosylation of paired helical filaments (PHFs) in AD brains was also detected by lectin binding [86]. Further studies showed that the maintenance of the paired helical filament structure is related to the *N*-glycosylation of tau [87]. 

In contrast to *N*-glycosylation, *O*-GlcNAcylation protects against aberrant hyperphosphorylation of tau protein in AD [50]. Tau in human brains was found to undergo *O*-GlcNAcylation on Ser and Thr residues, which competed with hyperphosphorylation. As hyperphosphorylation and *O*-GlcNAcylation occur reciprocally, decreased *O*-GlcNAcylation may precede the hyperphosphorylation of tau in AD brains [88]. Multiple *O*-GlcNAcylations have been detected on tau, and the level of *O*-GlcNAcylation of tau is decreased in AD brains compared to controls [89]. 

In addition, AGEs accumulation was detected in AD pathological deposits such as amyloid plaques [90]. Combined with the fact that patients with type 2 diabetes have higher risk of developing AD [91] and the connections of the insulin pathway with dementia [92], this observation further supported the hypothesis of considering AD as “type 3 diabetes” [93]. Moreover, AGEs upregulate the expression of BACE1 and Sirt1 expression via reactive oxygen species (ROS) [94], and clinical experiments indicated high levels of AGEs could influence the functional mobility in the aged population [95].

### 3.2. Glycans in Parkinson’s Disease 

PD is the second most common NDs and affects millions of people aged over 65 worldwide [62]. However, there is no accurate diagnostic marker. 

Pathologically, PD is characterized by the presence of Lewy bodies (α-synuclein aggregates), and the progressive loss of dopaminergic neurons [96]. Dopaminergic neuron loss-induced reduction of dopamine is considered to be the cause of motor defects in PD. Protein misfolding and aggregation, impairment of protein clearance pathways, energy failure, oxidative stress, and cell-autonomous mechanisms have all been shown to contribute to triggering and progression of dopaminergic neuronal loss in PD [97].

Glycan changes also occur in PD. Glycans are involved in neural cell signaling, cell death, and immune responses, all of which play important roles in PD pathogenesis [98]. Tri- and tetra-antennary glycan levels were altered in male PD patients, while the accumulation of *O*-linked glycosylation of α-synuclein (α-Sp22) was found in PD patient brains [98]. Furthermore, animal models deficient in sialic acid-containing ganglioside display PD-like symptoms, and administration of L-DOPA or cell-permeable ganglioside mimetics reversed these symptoms [6]. In addition, the evidence have proved the co-localization of AGEs and α- synuclein, which accelerate the aggregation of the protein, and the expression of RAGE was also found in PD patients [98].

The accumulation of aggregated α-synuclein is believed to be a vital contributor to PD pathogenesis. Emerging evidence suggests that migration of toxic α-synuclein between cells may propagate the disease [99]. α-synuclein can also bind to *N*-linked glycans to the surface of cells [100]. Further, cleavage of extracellular *N*-linked glycans, but not other carbohydrates, reduced α-synuclein cellular internalization, and a neuronal glycoprotein neurexin-1β can mediate glycan-dependent α-synuclein uptake [100]. GM1 is expressed in an age-dependent manner, and significant GM1 deficiencies have been observed in nigral dopaminergic neurons from PD patients [101]. Importantly, GM1-deficient animals display Parkinson-like symptoms that were alleviated by administration of LIGA-20 (a blood–brain barrier-permeable GM1 analogue) [102] Thus, GM1 ganglioside deficiency is believed to be one of the triggers of PD, suggesting that GM1 can be a potential therapeutic target for PD treatment.

### 3.3. Glycans in Huntington’s Disease 

As the most dominantly inherited brain disorder, HD is characterized by progressive neurodegeneration of striatal and cortical neurons, which leads to cognitive and motor dysfunction, behavioural disturbances, brain atrophy, bodyweight loss, and shortened lifespan [62]. The major pathological feature of HD is the presence of intracellular aggregates of mutant huntingtin protein (mHtt) [103]. The normal huntingtin gene encodes a huntingtin protein containing approximately 34 glutamine-coding (CAG) repeats. Extension of CAG repeats in the gene-encoding huntingtin results in an abnormal protein, which gradually aggregates and induces cell damage and causes deleterious effects in neuronal cells [104]. Altered expression of glycosyltransferase-encoding genes causes abnormal ganglioside metabolism in HD transgenic mice, as well as HD patients [105]. Glycoblotting and MALDI-TOF mass spectrometry analyses have found that the total glycome expression levels is considerably different between HD transgenic and control mice [106]. Changes in glycans have been observed in HD transgenic animals: increased core-fucosylated *N*-glycans were detected in the brain; increased sialylated biantennary type glycans and bisecting GlcNAc type glycans were detected in the serum; and decreased core 1-type *O*-glycans were detected in the serum, while core 2 type *O*-glycans were not detected. It was also found that glycosphingolipid GD1a was increased in the brain; and GM2-NeuGc was decreased in the serum [107]. Further, inhibition of *O*-GlcNAcylation also stimulates autophagy and reduces the huntingtin aggregates leading to enhanced neuronal cell viability [108]. In addition, the expression of RAGE is also observed in both astrocytes and neurons in caudate nucleus (CN) of HD patients [109].

### 3.4. Glycans in Multiple Sclerosis 

MS is an acquired chronic neurological disease affecting young adults [62]. MS affects nerve impulses in the spinal cord and optic nerves, including vision loss, fatigue, pain, and impaired coordination [110]. MS is characterized by inflammatory demyelination of axons and neurodegeneration. Through the regulation of inflammation, glycans have been associated with MS. In mice, *N*-glycan branching is required to prevent several pathologies characteristic of MS, including T cell hyperactivity, spontaneous inflammatory demyelination, and cytotoxic T lymphocyte antigen 4 (CTLA-4) endocytosis [111]. Absence of GlcNAc branching in neurons induces apoptosis and promotes T cell-mediated demyelination and autoimmunity, suggesting that GlcNAc-branching deficiencies may induce neurodegeneration in MS [112]. These findings suggest that targeting *N*-glycan biosynthesis is a possible therapeutic strategy against MS. In addition, an upregulation of both AGE and RAGE in patients with MS has been reported [113]. In contrast, soluble RAGE (sRAGE) decreased in the plasma of MS patients [114]. There are several forms of sRAGE and it has been reported sRAGE can antagonize RAGE signaling and ameliorate the deleterious effects of RAGE [31].

### 3.5. Glycans and Amyotrophic Lateral Sclerosis

ALS is a disorder of voluntary muscular movements. As a progressive disease, ALS starts with muscle stiffness, muscle twitching and weakness in limbs, and further weakness due to decreased muscle size. To date, there is no cure for this fatal ND. Altered expression levels of glycoproteins was detected in the sera or CSF of ALS patients [115]. Similarly, low levels of fucosylated glycans and high levels of sialylated glycans were detected in the serum of ALS patients [115]. Also, studies indicated CSF IgG *N*-glycosylation as a potential biomarker for ALS. Furthermore, a carbohydrate sulfotransferase, GlcNAc6ST1, is upregulated and identified as one of the top 40 ALS relevant genes in microglia [116]. In addition, the concentration of CML, one of the prominent AGEs, significantly elevated in the CSF of ALS patients [117]. Moreover, the levels of sRAGE are considerably lower in the serum of ALS patients [117].

## 4. Glycan-Based Therapies for Neurodegenerative Disease

As described, glycans have diverse functions from cellular recognition to organism development and disease progression. Due to their structural complexity and diversity, developing glycan-based therapies is challenging. However, with the development of new tools and techniques, several glycan-based therapies have been developed. In particular, glycosylation modulators that affect the attachment of glycans can be powerful tools for developing glycan-based therapies. 

### 4.1. Glycosylation Modulators

As detailed above, aberrant glycosylation and glycation lead to dysfunctional proteins and abnormal cellular function in NDs. Therefore, rebalancing glycosylation provides an opportunity for drug development. Glycosylation is regulated by two main enzymes: glycosyltransferases and glycosidases. Therefore, modifying the activity of these enzymes could be an effective therapeutic strategy. Indeed, a large number of small molecular compounds can regulate glycosylation by modulating glycosyltransferases and glycosidases directly or indirectly. These modulators can be classified into three main types: (1) inhibitors targeting the metabolism of common glycan precursors or intracellular glycan transport in the ER or Golgi; (2) tunicamycin, which blocks the transfer of GlcNAc-1-P (N-acetylglucosamine-1-phosphate) from UDP-GlcNAc to dolichol-P, which decreases the formation of dolichol-PP-GlcNAc and finally entirely blocks glycoprotein glycosylation; and (3) plant alkaloids, which block *N*-glycosylation by reducing α-glucosidases and α-mannosidases, resulting in deficiency of mature *N*-glycans on the cell surface [118]. Many natural compounds have been classified into these three groups.

Natural compounds classified into the first-class may have pleiotropic effects on glycan assembly. Brefeldin A, a fungal metabolite, causes retrograde transport of Golgi components back to the ER [119]. It was shown that brefeldin A at 1 ug/mL inhibited axonal growth and induced neurotoxicity in cultured neurons [120]. 6-diazo-5-oxo-L-norleucine (DON), a glutamine antagonist, blocks fructose-6-phosphate amidotransferase [121] and inhibits the proliferation of lymphocytes which respond to infection to prevent the brain inflammation [122]. However, DON also affects other glutamine-related molecules; therefore, the non-specific side effects should be considered. [118]. 

Tunicamycin is a natural nucleoside antibiotic that was first isolated from *Streptomyces lysosuperificus*. Tunicamycin blocks glycosylation of glycoproteins and results in many misfolded proteins, which in turn triggers ER stress [123]. Studies indicate that the application of tunicamycin protected against ischemia–reperfusion (I-R)-induced brain injury [124]. Similar to tunicamycin, amphomycin is a naturally occurring lipopeptide, and was first identified in *Streptomyces canus*. Amphomycin prevents dolichol-P-mannose synthesis due to the generation of complexes with the carrier lipid dolichol-P [125]. It suggested that amphomycin can be a compelling inhibitor of dolichol-P-dependent glycosylation.

One typical type of plant alkaloid, polyhydroxy indolizidine alkaloid, exhibits potent glycosidase inhibitory activity. Castanospermine and swainsonine are two representatives of this kind of compound, which mimic transition state of polysaccharide hydrolysis [126]. Castanospermine is a natural alkaloid isolated from the black bean, which prevents the trimming of the glucose residue of *N*-linked glycans and causes the accumulation of glucosylated chains [127]. An in vitro study showed that treatment with castanospermine enhances neurite fasciculation [128]. However, another study showed that exposure of castanospermine induces ultrastructural changes in subcellular organelles associated with glycoprotein synthesis, packaging and secretion in cultured embryonic mouse cerebellar neurons [129]. Swainsonine is isolated from the plant *Swainsona canescens*. As a sugar analogue, swainsonine effectively inhibits lysosomal α-mannosidase, which causes lysosomal storage disease [130]. Swainsonine also possesses anti-metastatic, anti-proliferative, and immunomodulatory properties [131]. 

Another mannosidase inhibitor is kifunensin, an alkaloid originally isolated from *Kitasatosporia kifunense*, which specifically inhibits α-mannosidase I [132]. In vitro studies showed that Kifunensine blocks the linkage of GlcNAc in *N*-glycans branches, which further prevents the effects of GlcNAc on neurogenesis of neural stem and progenitor cells (NSPC) [133]. Moreover, several other plant alkaloids affect *O*-GlcNAc attachment, such as alloxan and streptozotocin [134]. As these compounds lack specificity, they cause oxidative stress, inflammation and toxicity.

As most of the natural compounds discussed above inhibit global glycosylation and lack target specificity, their therapeutic application is limited. More specific compounds are therefore required for effective glycan therapeutics.

### 4.2. Glycan Mimetics from Natural Products

Glycomimetics are a class of synthetic small-molecule compounds that have been developed based on advances in the functional understanding of glycan–protein and glycan–lipid interactions [135]. Compared with glycosylation modulators, glycomimetics have enhanced affinity, selectivity, and drug-like properties. For example, chronic treatment with thiamet G, a *O*-GlcNAcase inhibitor, reduces the hyperphosphorylated tau in rTg4510 mice [136]. However, glycomimetics still have limitations as drug candidates or biological probes. For example, in the most common class of glycomimetics, imino sugars, bound glycosides are unstable because of variable *N*, *O*-acetal function [137]. Furthermore, some glycomimetic synthesis reactions are tremendously complicated due to their natural valency, topology, and density of carbohydrate presentation [138]. 

Some natural compounds have also been identified as glycan mimetics (Table 2).

Natural products possess enormous structural and chemical diversity and therefore represent an excellent source of drugs. Furthermore, their long-term use as herbal medicines means that they have proven drug efficacy and safety at a lower cost than many synthetic compounds [139]. Here, we summarize a group of natural products (including semisynthetic compounds) that functionally mimic glycans, have proven neuroprotective functions, and may therefore form a novel class of therapeutics for NDs. These “natural glycomimetics” are classified based on the glycans they mimic including human natural killer-1 (HNK-1) [140], LewisX (LeX) [141], neural cell adhesion molecule L1 (L1CAM) [142], and polysialic acid (PSA) [143]. The glycan-mimicking properties of these compounds have been confirmed by competitive enzyme-linked immunosorbent assay (ELISA) using glycan antibodies. 

#### 4.2.1. Human Natural Killer-1 (HNK-1) Mimicking Natural Compound

HNK-1 was first identified as a marker of human natural killer cells [144]. As a glycan epitope, HNK-1 is always associated with sulfoglucuronylglycolipids and glycoproteins. It has been confirmed that HNK-1 is widely found in the CNS and PNS and participates in various neural functions, including myelination, neurite outgrowth, and synaptic regeneration after nerve injury [145]. Absence of HNK-1 results in brain dysfunction such as defective synaptic plasticity and spatial learning [146,147]. HNK-1 epitopes contain several *N*-glycan-associated recognition molecules such as neural cell adhesion molecule (NCAM) and P0, a glycoprotein of the immunoglobulin superfamily. In addition, HNK-1 is associated with chondroitin sulfate proteoglycans that improve neurite outgrowth and neuronal cell adhesion [148]. Similarly, interactions between HNK-1 and the high mobility group box 1 (HMGB1) protein regulate cell–cell recognition and neuronal migration. 

Ursolic acid (3β-hydroxy-urs-12-en-28-oic-acid) is a naturally occurring pentacyclic triterpenoid identified as having HNK-1 mimetic functionality. The HNK-1-like activity of UA was confirmed using a competition assay with HNK-1 antibody binding through ELISA [140]. UA was first identified in the epicuticular waxes of apples and is also found in diverse classes of plants such as *Rosmarinus officinalis* (rosemary), *Ocimum basilicum* (basil), and some fruits such as pears and prunes. UA has attracted considerable interest as a herbal medicine due to its low toxicity and favorable pharmacological activities. UA exhibits a variety of biological functions such as anti-inflammatory [149], anti-oxidative [150], and neuroprotection [151] properties. For example, UA administration attenuates CCI4-induced hepatic dysfunction and protects against oxidative kidney damage by suppressing tumor necrosis factor alpha (TNF-α), interleukin 6 (IL-6), cyclooxygenase-2 (COX-2), nuclear factor kappa-light-chain-enhancer of activated B cells (NF-kβ), and signal transducer and activator of transcription 3 (STAT3) [152]. UA also exhibits strong neuroprotective activities by inhibiting inflammation and oxidative stress. For instance, UA can attenuate D-galactose-induced inflammatory responses in the mouse prefrontal cortex by suppressing advanced glycation of end-products [153]. UA also possesses the strong ability to inhibit ROS generation, suppress DNA fragmentation, and protect against Aβ–induced toxicity in PC12 cells [154]. Furthermore, UA successfully abolishes binding of Aβ and CD36 cells, thus preventing microglial activation and the production of cytokines and neurotoxins that may lead to AD [154].

#### 4.2.2. Lewis X (LeX) Mimicking Natural Compounds

Lewis X (LeX) is a trisaccharide usually attached to cell surface *O*-glycans. Association of Lewis X with secreted extracellular matrix (ECM) proteins was also observed. LeX belongs to the Lewis blood group antigens, a set of structurally related glycan moieties with fucosylated *N*-acetyllactosamine. In mammals, LeX regulates the proliferation of neural stem cells by activating Notch signaling and has further been confirmed as a neural stem cell marker [155]. Two modified forms of Lex, sulfoLeX and sialyl LewisX (sLeX), are involved in lymphocyte rolling and cancer metastasis [156]. However, there is little information regarding the function of sulfoLeX and sialyl sLeX in the nervous system.

Two natural compounds, gossypol and folic acid, have been identified as LeX mimetics [141]. Gossypol is a natural phenolic aldehyde first isolated from the cotton (*Gossypium*) plant of the family Malvaceae in 1899. Gossypol was first considered as a potential male contraceptive due to its strong anti-spermatogenic effects [157]. However, later studies revealed other potential therapeutic uses for gossypol including as an anti-tumor and neuroprotective agent. In breast cancer, gossypol kills tumor cells by modulating the expression of the cell cycle-regulatory proteins Rb and cyclin D1 [158]. In the nervous system, gossypol stimulates neurite outgrowth and regulates Erk signaling [159]. These findings suggest that gossypol might be a potential therapeutic agent in NDs. Folate occurs naturally in food, is a water-soluble B vitamin, and is considered a safe and effective compound. Folate is one form of folic acid mainly used for preventing and treating anemia caused by low blood folate levels (folate deficiency). A lack of folate during development may increase the risk of autism, leading to severe language delay and emotional problems [160]. Multiple studies have linked folate deficiency with dementia, poor cognitive function, and NDs. Melitta et al. found that folic acid promotes neuronal survival after hydrogen peroxide treatment [141]. Moreover, folic acid has been used as a supplement for pregnant women to reduce the chance of the neural tube defects (NTDs) in babies [161].

#### 4.2.3. L1CAM Mimicking Natural Compound

The neural cell adhesion molecule L1 (L1CAM) is a cell surface glycoprotein. As a member of the immunoglobulin supergene family of cell adhesion molecules, L1CAM is abundantly expressed in the nervous system and exerts a wide range of biological activities during brain development [162]. L1CAM is involved in neurite outgrowth and fasciculation through regulating cell adhesion and migration [163,164]. In disease, L1CAM enhances neuronal survival and stimulates axonal regeneration to improve behavioral outcomes [165]. Similarly, L1CAM overexpression enhances locomotor recovery after spinal cord injury. In contrast, mutations in L1CAM cause several disorders such as X-linked hydrocephalus with stenosis of the aqueduct of Sylvius (HSAS), rare X-linked recessive neurological disorder on the L1 disorder spectrum (MASA syndrome), and spastic paraplegia, also referred to as the “L1 syndromes”. However, the pathological mechanisms leading to L1 syndromes remain unclear.

Honokiol is a naturally occurring biphenolic compound extracted from the Magnolia tree and is widely used in traditional Asian medicine. Honokiol is a small molecule compound with high bioavailability, as it can cross the blood–brain barrier and the blood–cerebrospinal fluid barrier [166]. Moreover, honokiol possesses potent pharmacological activities, including anti-oxidative, anti-inflammatory, anti-tumorigenic, and neuroprotective properties [167]. Honokiol has been used as a therapeutic agent in the cardiovascular, gastrointestinal, and nervous systems, and the neuroprotective function of honokiol has been proven in several studies [168,169,170,171]. For instance, as a L1CAM mimetic, mice treated with honokiol showed locomotor recovery after spinal cord injury in an L1CAM-dependent manner [142]. Similarly, pre-clinical investigations demonstrated that the application of honokiol alleviates the effects of seizure and stroke and improves learning and memory in behavior models. Several pathways are implicated in the neuroprotective function of honokiol, including oxidative stress pathways and inhibition of inflammation. For example, honokiol reduces inflammatory factor production in glial cells by inhibiting NF-κB activation to further suppress the production of NO and TNF-α [172]. Hoi et al. also found that honokiol significantly reduced Aβ-induced neuronal death [173]. Moreover, the neuroprotective effects of honokiol in Aβ toxicity are related to the inhibition of caspase-3 activity, suppressed intracellular calcium elevation, and decreased ROS production [173].

#### 4.2.4. Polysialic Acid (PSA) Mimicking Natural Compounds

Polysialic acid is a glycan that predominantly binds to the NCAM. PSA consists of a linear polymer of sialic acid and is well-known for its role in the developing nervous system. PSA regulates various neuronal functions such as axon guidance, cell migration, differentiation, and cytokine responses [174]. PSA also mediates the interaction between NCAM and other molecules such as heparan sulfate proteoglycans. Recent studies have shown that differential ablation of polysialytransferases (ST8Sia IV (PST) and ST8Sia II (STX)) of PSA causes significant defects in axon growth and perinatal death in a mouse model [175].

Vinorelbine, a semisynthetic vinca alkaloid, is an Food and Drug Administration (FDA)-approved chemotherapy used to treat breast cancer and non-small cell lung cancer. As a microtubule destabilizing agent, vinorelbine stimulates mitotic spindle destruction and microtubule depolymerization at higher concentrations [176]. However, at lower concentrations, it can block mitotic progression at the G2-M phase [177]. Recent studies also indicate that vinorelbine can functionally mimic PSA and promote neurite outgrowth through regulation of myristoylated alanine-rich C kinase substrate, NCAM, and fibroblast growth factor receptor via Erk signaling [143].

## 5. Conclusions

Sugars coat all cells in every organism and are estimated to be the most abundant organic molecule on Earth. Glycan modifications on glycoproteins and glycolipids in the CNS play critical functions in NDs. Aberrant glycans have been observed in most NDs and therefore rebalancing glycosylation is a promising therapeutic strategy.

Glycans are inherently complex and heterogeneous in biological systems, and glycan therapeutics remain a growing but largely unexplored area. Over the last few decades, the development of new technologies, from gel electrophoresis-based methods to high-resolution MS-based approaches, have offered excellent opportunities to reveal molecular events related to glycan function and explore their application in NDs therapeutics. 

The natural compounds ursolic acid, gossypol, folic acid, honokiol, and vinorelbine (semisynthetic) functionally mimic glycans and may be of benefit in NDs. These discoveries indicate that natural compounds represent a vast and diverse library with which to identify glycan function-mimicking compounds. Future studies in this area might open new avenues for NDs treatment.

## Figures and Tables

**Table 1:** Glycosylation in neurodegenerative diseases.

Diseases	Protein/Gene Products	Known Glycosylation Types	Glycosylation Sites (confirmed)		Functions/Comments
Alzheimer’s disease (AD)	APP	N-glycosylated	Asn467	Asn496	1. Defects in N-glycosylation prevent the transportation and secretion of APP 2. O-glycosylated APP decreases Aβ secretion 3. Increase in tyrosine-linked glycan on Aβ fragments has been identified in the CSF samples of AD patients
		O-GlcNAcylated (APP695)	Thr291	Thr292	
			Thr576		
		O-GlcNAcylated (APP770)	Ser597	Ser606	
			Ser611	Thr616	
			Thr634	Thr635	
			Ser662	Ser680	
	BACE-1	N-glycosylated	Asn153	Asn172	1. Bisecting GlcNAc modification of BACE-1 increases Aβ production
			Asn223	Asn354	
	Tau	N-glycosylated	Asn167	Asn359	1. N-glycosylation of Tau appeared to be responsible for the maintenance of the PHFs structure 2. Level of O-GlcNAcylation of Tau is decreased in AD brains
			Asn359		
		O-GlcNAcylated	Ser400	Thr123	
	Nicastrin	N-glycosylated	16 potential sites		1. Defects of O-GlcNAcylation decrease Aβ plaques 2. Function of N-glycosylated remains poorly understood
		O-GlcNAcylated	Ser708		
	PS	None			
Parkinson’s disease (PD)	α-synuclein	O-GlcNAcylated	Thr33	Thr44	1. Accumulation of O-linked glycosylation of α-synuclein was found in PD patients
			Thr54	Thr59	
			Thr64	Thr72	
			Thr75	Thr81	
			Thr87		
Huntington’s disease (HD)	huntingtin	O-GlcNAcylated	N/A		1. O-GlcNAcylation regulates clearance of mHtt 2. O-GlcNAcylation stimulates autophagy and reduces huntingtin aggregation
Multiple Sclerosis (MS)	TNF-α	N/A	N/A		1. Absence of GlcNAc brancing in neurons induces apoptosis and promotes demyelination 2. N-glycan branching is required to prevent T cell hyperactivity, cytotoxic T lymphocyte antigen 4 (CTLA-4) endocytosis, spontaneous inflammatory demyelination in MS pathology
Amyotrophic Lateral Sclerosis (ALS)	SOD1	N/A	N/A		1. CSF IgG N-glycosylation as a potential biomarker for ALS 2. Altered expression of glycoproteins in the sera or CSF were detected in ALS patients

AD, Alzheimer’s disease; APP, amyloid precursor protein; Aβ, amyloid beta; CSF, cerebrospinal fluid; GlcNAc, N-acetylglucosamine; BACE-1, β-site APP-cleaving enzyme 1; PHFs, paired helical filaments; PS, presenilin; PD, Parkinson’s disease; HD, Huntington’s disease; mHtt, mutant huntingtin; MS, multiple sclerosis; TNF-α, tumor necrosis factor alpha; ALS, amyotrophic lateral sclerosis; SOD1, superoxide dismutase 1; Asn, asparagine; Thr, threonine; Ser, serine.

**Table 2:** Structure of natural/semisynthetic glycan mimetics.

Glycan/Glycoprotein	Natural/Semisynthetic Glycan Mimetics	
Human natural killer-1 (HNK-1)	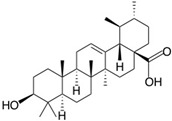	
	Ursolic acid	
Lewis X (Lex)	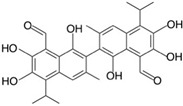	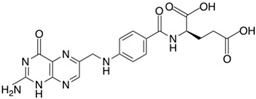
	Gossypol	Folic acid
Neural cell adhesion molecule L1 (L1CAM)	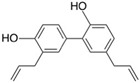	
	Honokiol	
Polysialic acid (PSA)	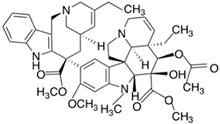	
	Vinorelbine	

## Abbreviations

AD	Alzheimer’s disease
AGEs	Advanced glycation end-product
AICD	Amyloid precursor protein intracellular domain
ALS	Amyotrophic lateral sclerosis
ANP	Atrial natriuretic peptide
APP	Amyloid precursor protein
Asn	Asparagine
Aβ	Amyloid beta
BACE-1	β-Site APP-cleaving enzyme 1
CAG	Glutamine-coding
CCI4	Carbon tetrachloride
CMD	Congenital muscular dystrophy
CML	*N*-carboxymethyl-lysine
CN	Caudate nucleus
CNS	Central nervous system
COX-2	Cyclooxygenase-2
CSF	Cerebrospinal fluid
CTF	C-terminal fragments
CTLA-4	Cytotoxic T lymphocyte antigen 4
dolichol-P	Dolichol-phosphate
DON	6-Diazo-5-oxo-L-norleucine
ECM	Extracellular matrix
ELISA	Enzyme-linked immunosorbent assay
ER	Endoplasmic reticulum
ERAD	ER-associated degradation
ErkFDA	Extracellular signal-regulated kinasesFood and Drug Administration
GlcNAc	*N*-acetylglucosamine
GlcNAc-1-P	*N*-acetylglucosamine-1-phosphate
GlcNAc6ST1	A carbohydrate sulfotransferase
GM1	Monosialotetrahexosylganglioside
GM2	Monosialic ganglioside
GM3	Monosialodihexosylganglioside
GSLs	Glycosphingolipids
HD	Huntington’s disease
HMGB1	High mobility group box 1 protein
HNK-1	Human natural killer-1 glycan antigen
HSAS	X-linked hydrocephalus with stenosis of the aqueduct of sylvius
I-R	Ischemia–reperfusion
IgG	Immunoglobulin G
IL-6	Interleukin 6
L-DOPA	Levodopa
L1CAM	Neural cell adhesion molecule L1
LacCer	Lactosylceramide
Lewy bodies	α-Synuclein aggregates
LeX	LewisX
LIGA-20	A blood–brain barrier-permeable GM1 analogue
MALDI-TOF	Matrix-assisted Laser Desorption/Ionization
MASA syndrome	A rare X-linked recessive neurological disorder on the L1 disorder spectrum
MEB	Muscle–eye–brain disease
mHtt	Mutant huntingtin
MS	Multiple Sclerosis
NCAM	Neural cell adhesion molecule
NeuGc	*N*-glycolylneuraminic acid
NF-kβ	Nuclear factor kappa-light-chain-enhancer of activated B cells
NO	Nitric oxide
NSPC	Neural stem and progenitor cells
NTD	Neural tube defect
*O*-GlcNAc	*O*-linked β-*N*-acetylglucosamine
OGA	*O*-GlcNAcase
OGT	*O*-GlcNAc transferase
P0	Myelin protein zero
p3 peptide	3-kDa fragments of amino-terminal truncated Aβ peptides
PD	Parkinson’s disease
PHFs	Paired helical filaments
PNS	Peripheral nervous system
pRb	Etinoblastoma protein
PS	Presenilin
PSA	Polysialic acid
PST	ST8Sia IV
PTM	Post-translational modification
RAGE	Receptor for advanced glycation end-products
ROS	Reactive oxygen species
Ser	Serine
Sirt1	NAD-dependent deacetylase sirtuin-1
sLeX	Sialyl LewisX
SOD1	Superoxide dismutase 1
sRAGE	Soluble receptor for advanced glycation end-products
ST	Sialyltransferase
STAT3	Signal transducer and activator of transcription 3
STX	ST8Sia II
Thr	Threonine
TNF-α	Tumor necrosis factor alpha
UA	Ursolic acid
UDP	Uridine diphosphate
α-Sp22	A glycosylated form of α-synuclein
